# Editorial: Clinical application of proteomics in kidney diseases

**DOI:** 10.3389/fmed.2022.965083

**Published:** 2022-07-14

**Authors:** Martin Pejchinovski, Pedro Magalhães, Jochen Metzeger

**Affiliations:** Department of Biomarker Discovery and Clinical Proteomics, Mosaiques Diagnostics and Therapeutics AG, Hanover, Germany

**Keywords:** proteomics, biomarkers, kidney diseases, diagnosis, prediction

Chronic kidney disease (CKD) remains to be one of the highest public health burdens worldwide with high-prevalence among the general population. The heterogeneity of the underlying disorders and stages of progression, as well as the lack of clear symptoms makes the whole healthcare process challenging not only for treatment but also for proactive and preventive care of the patients (1).

Currently available diagnostic markers are in many cases only indicative for late CKD stages and are not significantly superior in their accuracy to the estimation of the glomerular filtration rate (GFR), urine albumin to creatinine ratio (ACR) or immunohistochemically assessment of kidney biopsies. Although identification and characterization of various biomarkers (proteins/peptides, miRNA) in different body fluids using high-resolution techniques for biomarker-guided therapies have been a major focus for the clinical research in the last years, globally up to 16% of the people with a high-risk of CKD development are potentially unprotected (1–3). Therefore, the scope of the present Editorial “*Application of Proteomics in Kidney Diseases”* is to emphasize the importance of the identification and investigation of new and more reliable biomarkers that likely will improve early and accurate diagnosis of CKD, especially in context of improved patient stratification and prediction of disease outcome or treatment response. We are grateful to all of the authors for their valuable contribution to the present Research Topic and for the willingness to share some insights of their expertise with the research community to open new avenues for further improvement of CKD management.

Until to date, progress have been made for the application of proteomics in CKD. Development of biomarkers and identification of appropriate therapeutic targets are important aspects where scientists/clinical experts have put their focus on. In fact, early detection and prognosis of CKD including differential diagnosis and monitoring of drug treatment are areas with the greatest clinical need. Along these lines, Li et al. in their well-structured *review* have highlighted the role of Heme oxygenase-1 (HO-1), an enzyme involved in the process of heme degradation, in providing beneficial cytoprotective properties in cell metabolism. The authors outlined the involvement of HO-1 in regulation of oxidative stress and pathogenesis in various renal diseases. They provide a comprehensive overview of multiple studies elucidating the enzyme's function in metabolic processes for cell proliferation and cell maturation. Great emphasis was also laid to the description of therapeutic strategies where HO-1-overexpressing macrophages are used for treatment of kidney diseases.

Current clinical markers like deceased GFR function and/or presence of proteinuria are commonly considered as a gold standard for CKD diagnosis and its progression (4). To improve our moderate understanding on disease progression, it is necessary to have deeper insights into the mechanisms likely responsible for glomerular dysfunction and how podocyte epithelial cells are involved in this pathophysiological process. For this reason, not only expression of the podocyte cytoskeletal proteins synaptopodin, nephrin and podocin but also increased mutation rates and dysregulation of ion channels are crucial for altered kidney function making these observations ideal starting points for further investigations (5). In this respect, Zhou et al. have studied the effects of Transient receptor potential (TRP) C5 channel inhibition in puromycin aminonucleoside (PAN)-treated rats, in human iPSC-derived podocytes, and in kidney organoids. The authors were able to confirm that TRPC5 inhibition throughout AC1903 blockage in PAN-induced nephrosis rats protects podocyte cytoskeletal proteins from degradation and decrease levels of proteinuria. In a similar manner, inhibition of TRPC5 channels in 2D and 3D iPSC and organoid cell cultures altered the effect PAN-induced injury in human podocytes, providing evidence for targeting TRPC5 channels in the development of new potential CKD therapeutic strategies for patient care.

The potential application of two clinically important biomarkers for disease progression in IgA nephropathy has been investigated in a retrospective study with 2,153 patients by Shen et al. Evaluated levels of urinary β2-microglobulin (β2-MG) and Retinol-binding protein (RBP) have been assessed using restricted cubic splines and Cox proportional hazards models. As a result, the authors demonstrated that baseline levels of β2-MG and RBP were correlated with proteinuria, eGFR and histopathological Oxford classification IF/TA T scores. It was found by the authors that higher levels of β2-MG and RBP are indicative for worse outcome and disease progression in IgA nephropathy, highlighting the importance and prognostic utility of such non-invasive biomarkers in a clinical setting. Using a similar approach, Zhang et al. examined in a case-control study 6 potential plasma biomarkers that might be of clinical value in the prediction and patient stratification of acute kidney injury (AKI) progression after cardiac surgery. Out of the 6 investigated biomarker candidates, three demonstrated superior predictive capabilities and were further validated in an independent patient cohort. A marker panel consisting of these 3 markers, namely growth differentiation factor 15 (GDF15), soluble suppression of tumorigenicity 2 (ST2, IL1RL1), and soluble urokinase plasminogen activator receptor (uPAR) outperformed current clinical parameter models in their predictive performance.

The underlying cause of a kidney disease is from patient to patient highly variable. Some of the patients could have rare genetic mutations mediating slow growth of the kidneys, ultimately leading to late phase of disease progression often without possibility of any intervention. Autosomal recessive polycystic kidney disease (ARPKD) is one of them which is a heterogeneous genetic disease of the kidneys mainly affecting infants and children (6). It is characterized by the mutation of the polycystic kidney and hepatic disease 1 (PKHD1) gene causing massive enlargement of the kidneys following with episodes of kidney failure (7). Xu et al. reported a rare case of ARPKD where two siblings harbored biallelic variants in *PKHD1* (c.7205G>A and c.7973T>A) were identified. In their clinical case study, pathological analysis showed several well-known transformations of the renal tubules and collecting ducts. In addition, urinary exosome analysis confirmed decreased expression of proteins from the mitochondrial oxidative phosphorylation system (OXPHOS) which is involved in glycolysis, but also increased expression of lysosomal N-sulfoglucosamine sulfohydrolase (SGSH). Altogether, these findings provide new insights into the pathophysiology of the polycystic kidney caused by PKHD1 deficiency. Another extremely rare but clinical important disease is urological malakoplakia. It is characterized by the absence of any symptoms and induced by pathophysiological alterations in the phagocytotic system. Malakoplakia affects any part of the human body but most frequently occurs in the urinary system. In a clinical case study, Wang and Ren provided some insights into disease etiology with specific emphasis on the complexity of its diagnosis and distinction from infections, tuberculosis, as well as benign and malignant tumors of the urinary tract. The diagnosis and pathogenesis of malakoplakia is closely related to chronic infection and an impaired immune system. Although the authors suggested several clinical parameters for consideration, it was outlined in their report that the disease is mainly diagnosed by pathological examination. However, it should be emphasized that patients with such clinical manifestation are encouraged to visit their physicians more frequently in order to better elaborate the disease background.

Over the past decades, proteomic methodologies as well as novel laboratory technology have been proven to play a key role in providing important molecular information about disease processes. Knowing the fact that proteome profiles can reveal altered molecular pathways in CKD on top of the clinical/histopathological assessment, it is evident that their implementation in daily clinical routine is of unmet need. Certainty, clinical studies dealing with application of new drug targets supported by the biomarkers to predict and monitor CKD disease progression is expected to increase in the next years.

## Author contributions

All authors listed have made a substantial, direct, and intellectual contribution to the work and approved it for publication.

## Conflict of interest

MP, PM, and JM were employed by Mosaiques Diagnostics and Therapeutics AG.

## Publisher's note

All claims expressed in this article are solely those of the authors and do not necessarily represent those of their affiliated organizations, or those of the publisher, the editors and the reviewers. Any product that may be evaluated in this article, or claim that may be made by its manufacturer, is not guaranteed or endorsed by the publisher.
